# Hypergammaglobulinemia in the pediatric population

**DOI:** 10.1186/1546-0096-10-S1-A111

**Published:** 2012-07-13

**Authors:** Mindy S Lo, Robert P Sundel

**Affiliations:** 1 Children's Hospital Boston, Boston, MA, USA

## Purpose

Hypergammaglobulinemia in adult patients is usually related to malignancy, autoimmune disease, or infection. The differential diagnosis of hypergammaglobulinemia in children has not been similarly well defined. Malignancies and autoimmunity are much less common in children compared to the adult population, and therefore the implications of an elevated immunoglobulin in a pediatric patient are less clear. We therefore sought to establish the differential diagnosis of hypergammaglobulinemia in children through a retrospective cohort analysis.

## Methods

The Children’s Hospital Boston laboratory database was queried for all in- and out-patients with IgG levels ≥2000 mg/dL measured from 2000-2009. Charts were reviewed and patient characteristics, associated laboratory findings, diagnoses and outcomes were extracted. A random sampling of 10% of the patients was analyzed in detail.

## Results

A total of 1519 instances of IgG levels ≥2000 mg/dL belonging to 748 individual patients were identified. Of these, 85 patients were analyzed in detail. Thirty-one (36.5%) were excluded because they had received IVIG within one month prior to the abnormal test result. Indications for IVIG included Kawasaki disease (18 cases), immunodeficiency (3 cases), and autoimmune conditions (6 cases, including 2 patients who had received IVIG after rituximab-induced hypogammaglobulinemia). Among the remaining 54 patients who had not received IVIG, autoimmune/autoinflammatory conditions comprised the largest group (25 patients, 46.3%); cystic fibrosis was next most frequent (13 cases, 24.1%), followed by acute infection (9 patients, 16.7%) and malignancy (3 patients, 5.6%). The most frequent rheumatologic conditions were lupus (7 patients, 13.0%; 5 with SLE, 1 discoid lupus, and 1 minocycline-induced lupus), polyarticular JIA (4 cases, 7.4%) and MCTD (2 cases, 3.7%). Among infectious conditions, 6 patients had acute bacterial infections while 3 patients were presumed to have a self-limited viral process.

**Table 1:** Disease categories presenting with hypergammaglobulinemia

Diagnosis	Mean IgG	Meadian IgG	Number (%)
Autoimmunity	2458	2200	25 (46.3)
Lupus/SLE	2693	2400	7 (13.0)
Poly JIA	2321	2337	4 (7.4)
MCTD	2230	2230	2 (3.7)
Cystic fibrosis	2147	2120	13 (24.1)
Infection	2554	2330	9 (16.7)
Malignancy	2512	2188	3 (5.6)

## Conclusion

Among a cohort of patients at a large tertiary care children’s hospital with IgG level ≥2000 mg/dL, rheumatologic conditions constituted a much more frequent cause than infections. A wide variety of autoimmune diseases were represented, although not surprisingly lupus was the most frequent diagnosis. Interestingly, our cohort also included a large number of cystic fibrosis patients. The reason for hypergammaglobulinemia in many patients is not clear, though further studies may allow distinction between endogenous and exogenous lymphocyte stimulation.

## Disclosure

Mindy S. Lo: None; Robert P. Sundel: None.

